# Translation, Cultural Adaptation, and Psychometric Properties of the Danish Version of the Anti-Clot Treatment Scale

**DOI:** 10.1055/s-0038-1670631

**Published:** 2018-09-13

**Authors:** Willemijn J. Comuth, Henrik H. Lauridsen, Steen D. Kristensen, Anna-Marie B. Münster

**Affiliations:** 1 Department of Clinical Biochemistry, Regional Hospital of West Jutland, Herning, Denmark; 2 Department of Cardiology, Regional Hospital of West Jutland, Herning, Denmark; 3 Faculty of Health, Institute of Clinical Medicine, Aarhus University, Aarhus, Denmark; 4 Department of Sports Science and Clinical Biomechanics, University of Southern Denmark, Odense, Denmark; 5 Department of Cardiology, Aarhus University Hospital, Aarhus, Denmark; 6 Unit for Thrombosis Research, Department of Regional Health Research, University of Southern Denmark, Esbjerg, Denmark; 7 Department of Clinical Biochemistry, Hospital of South West Denmark, Esbjerg, Denmark

**Keywords:** atrial fibrillation, cardiology, psychological distress, thrombosis

## Abstract

**Background** The Anti-Clot Treatment Scale (ACTS) is a 17-item, 2-factor (Burdens and Benefits), patient-reported outcome instrument to evaluate patient satisfaction with oral anticoagulant treatment.

**Objectives** This study aimed to translate and culturally adapt the English version of the ACTS into Danish and to subsequently validate the Danish version in a population of patients treated with dabigatran etexilate for atrial fibrillation.

**Methods** The ACTS was translated into Danish and culturally adapted. This prospective phase 4 study included 232 respondents who completed the Danish ACTS after 1 month of treatment with dabigatran etexilate for atrial fibrillation. Psychometric properties were evaluated. For test–retest reliability, the ACTS was measured twice, 2 weeks apart, in a subgroup of 50 stable patients.

**Results** Generally, a high level of treatment satisfaction was found. Confirmatory factor analysis showed a suboptimal fit for the two-factor model of the original version. Using modification indices of confirmatory factor analysis, a four-factor model had the best fit. Cronbach's α for internal consistency was acceptable at 0.78. There was good test–retest reliability with intraclass correlation at 0.80. Smallest detectable changes (SDCs) for individual patients were 5.89 points for the total ACTS, 5.57 for the reverse Burdens, and 3.34 for Benefits scores. Group SDCs were 0.39, 0.37, and 0.22 respectively. Substantial ceiling effects limit the ability to detect improvement at the high end of the scale.

**Conclusion** The Danish version of the ACTS has inadequate structural validity. Reliability was acceptable. Ceiling effects challenge detection of improvement of treatment satisfaction in clinical practice in this patient population.

## Introduction

Atrial fibrillation has a prevalence of 2 to 3% in the general population and more than 10% in those who are older than 80 years, making it the most common sustained cardiac arrhythmia. 1 2 Patients with atrial fibrillation have an increased risk of morbidity and mortality and long-term treatment with an oral anticoagulant for the prevention of thromboembolic complications is often indicated. 1 Oral anticoagulant treatment with vitamin K antagonists (VKAs) has several disadvantages which may negatively influence patient treatment satisfaction, including the need for frequent blood testing and dose adjustments, potential interactions with food and other drugs, and an increased risk of bleeding and bruising. 3 The increased bleeding risk of VKAs may limit physical activity and increase psychological distress. 4

Dabigatran etexilate is one of the non–vitamin K antagonist oral anticoagulants (NOACs) and has been available in Europe for the treatment of atrial fibrillation patients from 2011 onward. 5 Dabigatran etexilate is also approved for the prevention and treatment of venous thromboembolism. 6 It has lower risk of life-threatening and intracranial bleeding in comparison to VKAs 7 ; routine monitoring of blood tests measuring the anticoagulant effect is not required and interactions with other drugs are uncommon. 7 These characteristics of dabigatran etexilate are expected to improve treatment satisfaction in comparison to VKAs. 3 Factors that could negatively influence patient acceptability are the cost of the dabigatran etexilate, side effects, twice-daily dosing, and the relatively large size of the capsules.

Patient-reported outcomes (PROs) have gained importance in patient care and medical research over the past decades. 8 PROs are efficient, are inexpensive, help to improve patient care, identify areas where further development is needed, and enhance patient satisfaction with health care systems. 8

Secondary to the development of NOACs, a new PRO measurement instrument for evaluating their effectiveness on anticoagulant treatment satisfaction became available in 2006: the Anti-Clot Treatment Scale (ACTS). 9 The ACTS was developed in English and is a modification of the Duke Anticoagulation Satisfaction Scale (DASS), developed for VKA treatment. 9 It consists of 17 questions and 12 of these concern the negative aspects (Burdens), whereas 3 questions address the positive aspects (Benefits) of anticoagulant treatment. Additionally, two global questions regarding treatment satisfaction are included. 9 Validation of the ACTS in rivaroxaban-treated patients showed acceptable reliability and validity. 9 The ACTS has not been validated specifically in dabigatran etexilate–treated atrial fibrillation patients.

The ACTS is used in many recent and ongoing international studies and registries for the evaluation of treatment satisfaction, 10 11 12 13 also allowing comparison between different oral anticoagulants. The questionnaire can, however, only be used in countries where a translation in the national language is available. Currently, the ACTS is accessible in 10 different languages. 14 However, it has not previously been translated to Danish. 14

The aim of this study was to translate and culturally adapt the ACTS into Danish and to validate the ACTS in a population of Danish patients treated with dabigatran etexilate for atrial fibrillation.

## Materials and Methods

### Translation and Adaptation

Permission for use of the ACTS was acquired from Mapi Research Trust, user and translation agreements were signed by both parties. Linguistic validation and cross-cultural adaptation from English to Danish was performed according to the International Society for Pharmacoeconomics 15 Outcomes Research (ISPOR) Principles of Good Practice and Guidelines for the Process of Cross-Cultural Adaptation of Self-Report Measures 16 and Mapi Validation Guidelines, which are very similar.

Six phases of linguistic validation were conducted:

1. *Forward translation* . Two professional translators with Danish as mother tongue and English at bilingual level translated the ACTS independently from each other. Forward Translator 1 (FT1) was informed about the clinical problem and patient group involved; Forward Translator 2 (FT2) was not informed about this (naive translator).
2. *Reconciliation* . The two versions of the forward translations in Danish were combined (FT-12) after agreement within the study group about the best formulations where there were differences in translation between the two versions.
3. *Backward translation.* Two professional translators with English as their mother tongue and living in Denmark translated the combined Danish version (T-12) of the ACTS back to English (BT1 and BT2).
4. *Harmonization.* Discussion within the study group of the differences between the original and the backward translations took place. Adjustments in T-12 were made if found appropriate and a consensus version was created.
5. *Cognitive debriefing.* The consensus version was tested on 10 patients who were native speakers of the Danish language and who were treated with dabigatran etexilate for atrial fibrillation (pilot testing). A face-to-face interview was performed by one of the Danish in the study group, while another person in the study group observed the interview. We collected information on the characteristics of the patients, time taken to interview, patient-reported potential problems with the questionnaire, and possible solutions proposed by patients, as well as the patients' opinion about the questionnaire using a structured interview guide.
6. *Proofreading.* Fine-tuning of the wording of the questionnaire was performed to obtain the final version of the Danish ACTS.

Subsequently, the Danish version of the ACTS was submitted to the developers at Mapi Research Trust.

### Validation Study

We used the COSMIN taxonomy of measurement properties and definitions for health-related PRO outcomes 17 to evaluate the psychometric properties of the ACTS. The COSMIN checklist was used to ascertain that all information needed was reported, to enable an appropriate evaluation of the quality of this study and to facilitate uniform reporting of validation studies. 17 ACTS patient characteristics, validity, reliability, and floor and ceiling effects were examined.

#### Data Collection

This is a single-center prospective noninterventional phase 4 study performed between January 2015 and December 2017 in the outpatient clinic and cardiology ward at Herning Regional Hospital, Denmark. The study followed the principles outlined in the Declaration of Helsinki and was approved by the Research Ethics Committee for the Region of Mid-Jutland (case number 1–10–72–52–15) and the Danish Data Protection Agency (case number 1–16–02–191–14). All patients included in the study provided oral and written informed consent before inclusion. The trial was registered at ClinicalTrials.gov (Identifier: NCT 03280368).

The target population was patients 18 years of age or older, with newly diagnosed atrial fibrillation or atrial flutter and an indication for anticoagulant therapy with dabigatran etexilate.

At baseline, demographic data were collected. An initial follow-up visit was planned 1 month after initiation of dabigatran etexilate, during which patients filled out the ACTS. As the respondents filled out the questionnaires at the outpatient clinic and the ACTS was immediately checked for completeness by the healthcare workers present at the consultation, there were no missing items.

#### Outcome Measures

The ACTS is a two-dimensional (Burdens and Benefits) tool to assess patient satisfaction with anticoagulant treatment. The ACTS has been validated in patients treated with rivaroxaban for venous thromboembolism (VTE), 9 consistently satisfying traditional reliability and validity criteria across multiple language datasets. 9 In another validation study in Spanish atrial fibrillation patients treated with oral anticoagulants, it was also concluded that the questionnaire is valid, reliable, and feasible. 18 The article is published in Spanish, with an abstract available in English. In the abstract, it is not specified which oral anticoagulants patients were treated with. 18

The ACTS uses a Likert-like scale ranging from 1 to 5 for each item (1 = not at all, 2 = a little, 3 = moderately, 4 = quite a bit, 5 = extremely). The scale includes 15 items and two global questions (one Burdens and one Benefits question). There are 12 standard questions regarding Burdens (score range: 12–60) and 3 standard questions regarding Benefits (score range: 3–15). For the total ACTS score (range: 15–75), the scores for the Burdens are reversed (reverse Burdens score), so that a higher score means higher treatment satisfaction. The results for the global questions are not included in the total ACTS score.

#### Sample Size

Rules of thumb for sample size recommendations vary between 4 and 10 cases per item for factor analysis, with 100 patients as an absolute minimum. 19 Hereby the target sample size for factor analysis of the ACTS was 60 to 150 patients. The number of patients who completed the study was 232.

#### Psychometric Evaluation

##### Validity

Content validity was evaluated by an expert panel consisting of potential users of the questionnaire (patients, clinical cardiologists, cardiovascular researchers). It was assessed whether the ACTS was a good instrument to measure patient treatment satisfaction with anticoagulant treatment, with specific focus on dabigatran etexilate, based on the opinions of the potential users. The members of the expert panel rated each item on the scale according to its relevance to measure medication adherence to dabigatran treatment in atrial fibrillation patients. A Likert-type scale was used, ranging from 1 (“not relevant”) to 4 (“very relevant”). The item-level content validity index (I-CVI) and the scale-level content validity index (S-CVI) were subsequently calculated. I-CVI is the proportion of the experts rating the score of 3 (“relevant”) or 4 (“very relevant”). S-CVI is calculated as the mean of all I-CVIs. I-CVI greater than 0.80 and S-CVI greater than 0.90 are considered acceptable. 20

Structural validity was measured using confirmatory factor analysis (CFA) for the two-factor model of the original version of the ACTS. Subsequently, modification indices of CFA were used in an exploratory fashion to find the optimal model for the underlying dimensions in the Danish version of the ACTS. Models with one, three, and four factors were tested. To assess the fit of the models to the data, the comparative fit index (CFI), the root mean square error of approximation (RMSEA), the standardized root mean square residual (SRMR), and the Tucker–Lewis index (TLI) were used. Guidelines suggest that models with a CFI of close to 0.95 or higher, RMSEA close to 0.06 or lower, SRMR close to 0.08 or lower, and TLI close to 0.95 or higher are representative of good-fitting models. 19 21 A CFI value between 0.90 and 0.95 and a TLI value of 0.90 or higher indicate an acceptable model fit. 19 21

##### Reliability

Internal consistency was calculated using Cronbach's α. A Cronbach's α between 0.70 and 0.90 is indicative of good internal consistency. 19 Inter-item correlations (IICs) were measured for the Benefits and reverse Burdens scales. IIC for items within one dimension should be between 0.20 and 0.50. 19 If the correlation of two items is higher than 0.70, they measure almost the same thing and one of the items could be deleted. 19

Test–retest reliability was tested in a subgroup of 50 patients 19 who completed the questionnaire twice, 2 weeks apart, after at least 3 months of treatment with dabigatran etexilate. Agreement of repeated measurements was presented as Bland–Altman plots with 95% limits of agreement (LOAs). Measurement error was defined as 1.96 × SD _diff_ , where SD _diff_ equals the standard deviation of the differences between the two measurements, in case of negligible systematic differences. 19 The smallest detectable change for an individual patient (SDC _ind_ ) was defined as change outside the LOAs. 19 22 SDC for the group score (SDC _group_ ) was calculated as SDC _ind_ /√n. 19

One-way analysis of variance (ANOVA) was performed to calculate the intraclass correlation coefficient (ICC 2.1A). 23 An ICC is expressed as a value between 0 and 1. ICC values of least 0.70 are considered acceptable, but values of 0.80 or higher are desirable. 19

##### Floor and ceiling effects

Interpretability of the Danish version of the ACTS was evaluated by the distribution of the scores of the instrument, floor and ceiling effects, and subgroup analyses for gender and age. With regard to floor and ceiling effects, McHorney and Tarlov suggested that PRO instruments with more than 15% of respondents scoring the highest or lowest score initially should not be used. 19 24 However, as an observed change must be at least equal to the SDC to be 95% confident that the change is not simply due to measurement error, more than 15% of respondents scoring within the SDC at the upper or lower end of the scales more reliably indicates a floor or ceiling effect. 24 In this way, the SDC was used to evaluate floor and ceiling effects for the specific patient population included in the study. Scale width indicates the capacity of a scale to have initial scores that are far enough onto the scale to allow detection of change in scores over time. 24 To reliably detect change over time, there has to be sufficient scale width. Therefore, there should be room for improvement at the high end of the scale as much as the SDC and room for worsening of treatment satisfaction as much as the SDC at the lower end of the scale. 24

#### Statistical Analyses

Demographic results were presented as means and standard deviations (SD). Nonparametric results were expressed as medians and ranges. Shapiro–Wilks tests for normality were used in combination with histograms and QQ-plots of the measurement errors to evaluate normality. For demographic data, a two-tailed *t* -test was used to evaluate the difference in means between men and women. The subgroup analyses on ACTS results were performed for age and gender using Mann–Whitney–Wilcoxon tests. A *p* -value of less than 0.05 was considered statistically significant.

Statistical analyses were performed using Stata version 15 statistical software. SigmaPlot 13.0 and Lucidchart software were used for the graphical work.

## Results

### Translation and Adaptation

The English version of the ACTS was successfully translated into the Danish version. Forward and backward translators reported no major problems with regard to comprehension of the instrument. They had suggestions for minor improvements by use of more typically Danish phrases and words. These were incorporated into the final Danish version of the ACTS.

### Pilot Testing

Pilot testing of the Danish version of the ACTS was performed in 10 patients (40% men, mean age: 74.2 years; SD: 6.4) who had been treated uninterruptedly with dabigatran etexilate for atrial fibrillation for at least 4 weeks. The mean time to complete the ACTS was 7 minutes 45 seconds (SD: 4 minutes 30 seconds). Minor adjustments were made, such as in question 4: “How bothered are you by having to avoid other medicines (e.g., aspirin) as a result of your anti-clot treatment?” Here, the example of aspirin was removed, as concomitant use of aspirin and an oral anticoagulant no longer is a contraindication (even though caution should be exercised, especially in patients at high bleeding risk) according to guidelines. 25 In patients with acute coronary syndrome, for example, combination of dabigatran etexilate with antiplatelet medication is recommended. 25 No major problems were reported by the patients with regard to understanding the questions themselves. The ACTS was found to be acceptable by patients and feasible by the study group.

### Validation Study

#### Patient Characteristics

**Fig. 1:**
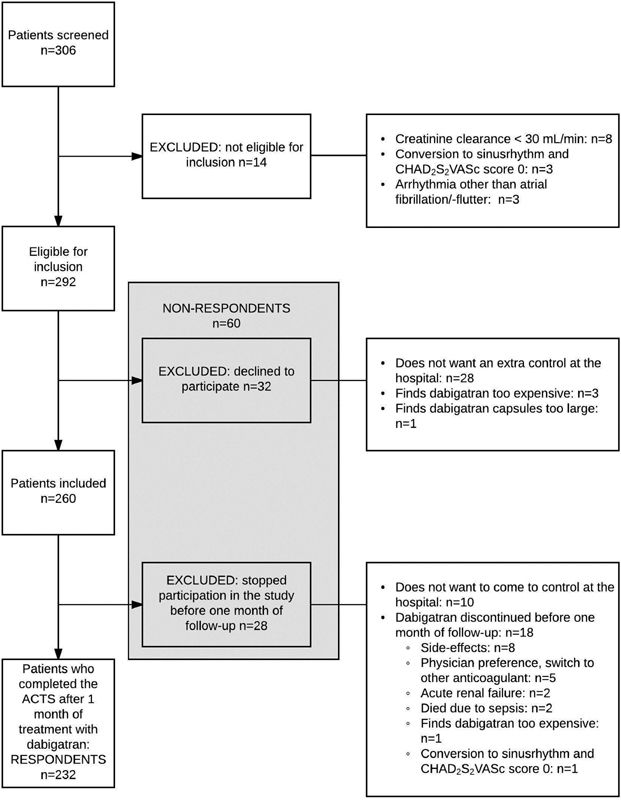
Flowchart of the study population.

Out of 306 patients screened, 292 patients had atrial fibrillation/flutter and an indication for dabigatran etexilate and were eligible for inclusion in the study. A total of 260 patients agreed to participate in the study. After 1 month of treatment with dabigatran etexilate, 232 patients (respondents) were still participating in the study and completed the ACTS. The 60 patients who were eligible for inclusion in the study but who did not want to participate or who did not complete follow-up at 1 month were defined as the nonrespondents. Fig. 1 shows a flow diagram of the study population.

Respondent and nonrespondent characteristics are shown in Table 1 . Nonrespondents had a statistically significantly higher age, lower weight and body mass index (BMI), as well as lower creatinine clearance, and as a consequence more often received the lower dose of dabigatran (110 mg twice daily) than respondents did. Nonrespondent females had a significantly higher CHA _2_ DS _2_ VASc score in comparison to respondent females. For males, there was no significant difference in CHA _2_ DS _2_ VASc scores. No differences were seen between respondents and nonrespondents with regard to HAS-BLED score and hemoglobin levels.

**Table 1:** Clinical characteristics of the study population

Characteristics	Respondents ( *n* = 232)	Nonrespondents ( *n* = 59)	*p* -Value
Gender ( *n* , %)			
Males	131 (56.5)	27 (45.0)	0.09
Females	101 (43.5)	33 (55.0)	
Age (y) a	69.8 (9.3)	75.8 (9.6)	<0.01
Males	68.6 (9.2)	74.0 (10.1)	0.03
Females	71.4 (9.2)	77.2 (9.0)	<0.01
Weight (kg) a	84.8 (18.3)	75.3 (16.3)	<0.01
Males	89.6 (18.1)	82.4 (16.4)	0.07
Females	78.5 (16.6)	69.8 (14.1)	<0.01
BMI (kg/m ^2^ ) a	28.2 (5.3)	26.3 (4.9)	0.02
Males	28.2 (5.0)	26.3 (5.1)	0.07
Females	28.1 (5.8)	26.3 (17.5)	0.11
CHA _2_ DS _2_ VASc score a	2.4 (1.3)	2.7 (1.3)	0.12
Males	2.0 (1.2)	1.8 (1.0)	0.24
Females	3.0 (1.2)	3.5 (0.9)	0.02
HASbled score a	1.0 (0.7)	1.3 (0.7)	0.06
Males	1.0 (0.7)	1.2 (0.8)	0.27
Females	1.1 (0.6)	1.3 (0.6)	0.15
Dabigatran dose (150 mg BID; *n* , %)	191 (82.3)	32 (53.3)	<0.01
Males	113 (86.3)	17 (63.0)	<0.01
Females	78 (77.2)	15 (45.4)	<0.01
Creatine clearance a (Cockroft, mL/min)	90.2 (30.8)	72.0 (28.3)	<0.01
Males	94.4 (29.2)	82.2 (29.6)	0.06
Females	84.7 (32.0)	64.0 (24.9)	<0.01
Hemoglobin (mmol/L) a	8.9 (0.8)	8.9 (0.8)	0.13
Males	9.1 (0.8)	9.1 (0.8)	0.07
Females	8.5 (0.8)	8.5 (0.8)	0.26

Abbreviations: BID, twice daily; BMI, body mass index.

a Mean value is shown, with standard deviation in brackets.

**Fig. 2:**
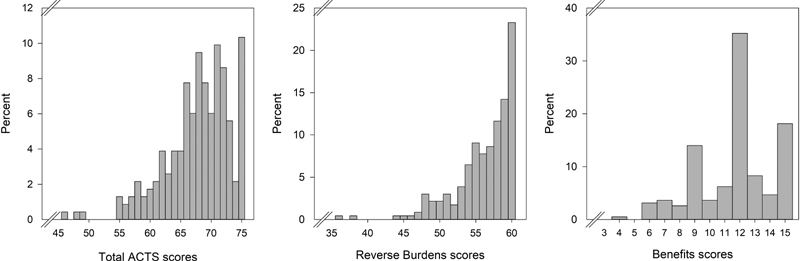
Distribution of the Anti-Clot Treatment Scale: total, reverse Burdens, and Benefits scores; *n* = 232 respondents at baseline.

The distributions of the ACTS total, reverse Burdens, and Benefits scores are presented in Fig. 2 . The distributions of the total ACTS (median: 69, range: 46–75), reverse Burdens (median: 57, range: 36–60), and Benefits scores (median: 12, range: 4–15) are left skewed.

Subgroup analysis for gender, age, CHA _2_ DS _2_ VASc score, and dabigatran dose are presented in Table 2 . Subgroup analysis showed no difference in ACTS scores for gender. Respondents aged 65 years and older had significantly higher total ACTS score and reverse Burdens scores, indicating higher treatment satisfaction, in comparison to younger patients ( *p* < 0.01). There was no significant difference in total ACTS, Burdens, or Benefits scores when using age 75 as a cutoff point. Respondents with a CHA _2_ DS _2_ VASc score of 2 or higher had a significantly higher total ACTS and reverse Burdens score compared with those with a CHA _2_ DS _2_ VASc score of 0 or 1 ( *p* < 0.01). For the Benefits score, there was no difference for age or CHA _2_ DS _2_ VASc score subgroups. A significantly higher Benefits score was seen on the lower dose of dabigatran (110 mg twice daily) in comparison to respondents treated with the standard dose (150 mg twice daily; *p* = 0.04), while there were no differences for the total ACTS and reverse Burdens scores.

**Table 2:** Subgroup analysis

Subgroup analysis	Groups	Total ACTS Median (range)	*p* -Value	Reverse Burdens Median (range)	*p* -Value	Benefits Median (range)	*p* -Value
Gender	Men	69 (48–75)	0.50	57 (38–60)	0.28	12 (4–15)	0.64
	Women	69 (46–75)		58 (36–60)		12 (6–15)	
Age (y)	<65	67 (46–75)	<0.01	55 (36–60)	<0.01	12 (6–15)	0.84
	≥65	69 (48–75)		58 (44–60)		12 (4–15)	
	<75	68 (46–75)	0.72	57 (36–60)	0.08	12 (4–15)	0.05
	≥75	69 (48–75)		58 (44–60)		12 (4–15)	
CHA _2_ DS _2_ VASc score	0 or 1	67 (49–75)	<0.01	55 (38–60)	<0.01	12 (6–15)	0.80
	≥2	69 (46–75)		58 (36–60)		12 (4–15)	
Dabigatran dose	150 mg BID	69 (48–75)	0.54	57 (36–60)	0.67	12 (4–15)	0.04
	110 mg BID	68 (46–75)		58 (44–60)		12 (4–15)	

Abbreviations: ACTS, Anti-Clot Treatment Scale; BID, twice daily.

Notes: Respondents aged 65 years and older and those with a CHA _2_ DS _2_ VASc score of 2 or higher had a significantly higher total Anti-Clot Treatment Scale score and reverse Burdens scores, indicating higher treatment satisfaction, in comparison to younger patients and those with a lower CHA _2_ DS _2_ VASc score, respectively. A significantly higher Benefits score was seen on the lower dose of dabigatran (110 mg twice daily) in comparison to respondents treated with the standard dose (150 mg twice daily).

### Psychometric Properties

#### Validity

The ACTS has good content validity. It was judged by our expert panel to give a more than adequate reflection of the construct “ *patient satisfaction with oral anticoagulant treatment* .” All items were scored with an I-CVI of 1.00, resulting in an S-CVI of 1.00.

**Fig. 3:**
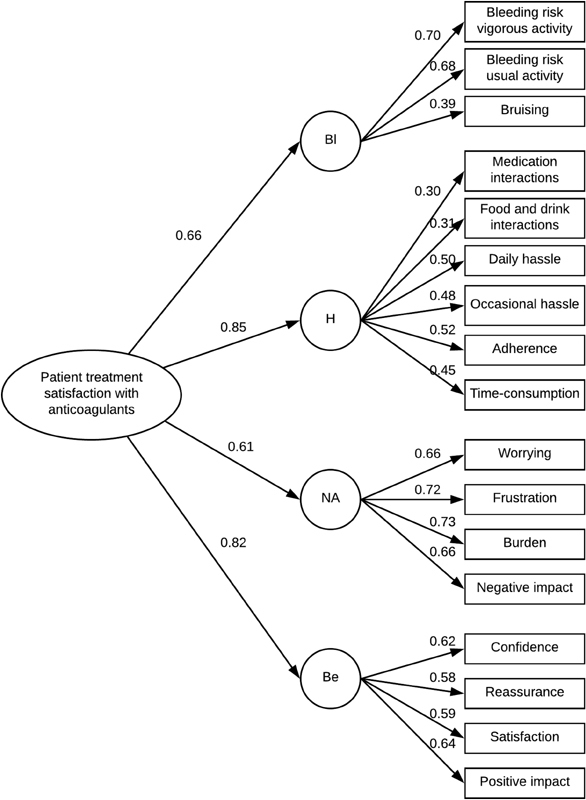
Optimal factor model. Factor analysis showed that a four-factor model had the best fit to the Danish version of the Anti-Clot Treatment Scale. The four domains were named Bleeding (Bl), Hassle (H), Negative affect (NA), and Benefits (Be). Standardized factor loadings are indicated for each parameter.

CFA of the two-factor model (Burdens and Benefits) resulted in the following fit indices: CFI of 0.82, RMSEA of 0.08, SRMR of 0.08, and TLI of 0.92. One of the criteria (SRMR) was fulfilled, but the others (CFI, RMSEA, and TLI) were not. This suggests that the Danish version of the ACTS does not have an adequate fit for the two-factor model of the original ACTS version. Exploration of one-, three-, and four-factor models revealed that the four-factor model had the best and acceptable fit indices for our data ( Table 3 ). The best-fitting model consisted of the factors Bleeding, Hassle, Negative affect, and Benefits ( Fig. 3 ).

**Table 3:** Fit indices for the two- and four-factor models

Model fit indices	CFI	RMSEA	SRMR	TLI
Two-factor model (Bl + H + NA, Be)	0.82	0.09	0.08	0.70
Four-factor model (Bl, I + H, NA, Be)	0.91	0.06	0.08	0.90

Abbreviations: Be, benefits; Bl, bleeding; CFI, comparative fit index; H, hassle; I, interactions; NA, negative affect; RMSEA, root mean square error of approximation; SRMR, standardized root mean square residual; TLI, Tucker–Lewis index.

Notes: Using modification indices of confirmatory factor analysis, it was found that the four-factor model, and not the two-factor model of the original version of the Anti-Clot Treatment Scale (ACTS), fitted best to the Danish version of the ACTS, with acceptable goodness-of-fit indices

#### Reliability

Cronbach's α was 0.80 for the Burdens scale and 0.83 for the Benefits scale, showing good internal consistency. So on average, the items measured the same construct. Average IICs of 0.19 and 0.22 were found for the total ACTS and the Burdens scores, respectively, which was within the predetermined acceptability criteria. The average IIC of the Benefits scale was 0.53, slightly higher than acceptable, indicating that items in this scale are very similar.

**Fig. 4:**
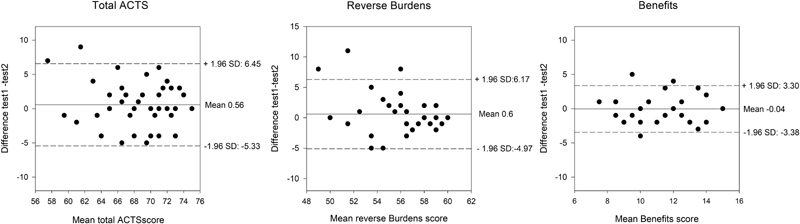
Bland–Altman plots for two measurements in stable patients. Solid lines indicate the mean difference, while the dashed lines indicate the 95% limits of agreement.

Bland–Altman plots with LOAs for the reverse Burdens and Benefits scales are presented in Fig. 4 . There were no signs of an important systemic difference and mean differences between the two measurements are close to zero. For the total ACTS score, the measurement error (SDC _ind_ ) was 5.89. The measurement error was somewhat larger for the reverse Burdens scale (5.57) than for the Benefits scale (3.34) in stable patients. Thus, relatively smaller changes can be identified as real changes in individual patients for the Benefits score than for the reverse Burdens score. SDC _groups_ were 0.39 for the total ACTS, 0.37 for the reverse Burdens, and 0.22 for Benefits.

The test–retest reliability was acceptable for the reverse Burdens and Benefits scales with ICC for both scales of 0.70 (95% confidence interval [CI]: 0.55–0.84). The total ACTS score showed good test–retest reliability with an ICC of 0.80 (95% CI: 0.70–0.90).

#### Floor and Ceiling Effects

None of the respondents scored the lowest possible score for treatment satisfaction on any of the scales. Using the SDC method, 0% of respondents had a total ACTS score of less than 18, 0% had a reverse Burdens score of less than 17, and 3.6% had a Benefits score of less than 7. Thus, no floor effects were seen.

A total of 10.3% of the respondents scored the maximum 75 points for treatment satisfaction on the total ACTS, which is within the 15% acceptance limit. 19 A ceiling effect was seen for the reverse Burdens score, with 22.8% of respondents scoring the highest score of 60. For the Benefits scale, a ceiling effect was also seen; 18.1% of respondents scored the maximum of 15 points. When using the SDC method for the calculation of ceiling effects, 24 substantial ceiling effects were seen across all scales; 43.1% of respondents had a total ACTS score of higher than 69, 57.8% had a reverse Burdens score of higher than 54, and 66.4% had a Benefits score of higher than 11. In these patients scoring at the high ends of the scales, potential improvement of treatment satisfaction over time could not be detected at an individual level and scale width was insufficient.

## Discussion

The ACTS was successfully translated into Danish, culturally adapted and validated in a group of 232 patients treated with dabigatran etexilate for atrial fibrillation. One of the main findings of the validation of the Danish version of the ACTS was that predetermined requirements for structural validity were not met. CFA of the Danish version of the ACTS fits did not fulfil all predetermined goodness of fit criteria for the two-factor model of the original English version of the PRO instrument. Instead, exploratory factor analysis suggests a better fit of the Danish ACTS version to a four-factor model. Good internal consistency and acceptable test–retest reliability were found. In our patient population, a high treatment satisfaction was seen, with ceiling effects of the total ACTS, reverse Burdens, and Benefits scores. This limits detection of improvement of treatment satisfaction over time in a large proportion of respondents at an individual level. As measurement error is reduced by a factor √ *n,* when a group of *n* patients is studied, changes can be detected reliably at group level when performing clinical research. Floor effects were not seen, so worsening of oral anticoagulant treatment satisfaction can potentially be detected. Generally, the total ACTS score and the Burdens score performed better than the Benefits score alone.

Cano et al originally developed and validated the ACTS to assess the burdens and benefits of anticoagulant therapy in patients with VTE. 9 The questionnaire satisfied traditional psychometric data quality, scaling, assumptions, targeting, reliability, validity, and responsiveness criteria. 9 Another study validated the ACTS in atrial fibrillation patients in Spain, and showed good reliability, validity, and feasibility. 18

Cano et al validated the ACTS in several languages in sample sizes similar to the one in our study. The patients in our study were older with a mean age of 70 years in comparison to 57 years in the VTE patients included in the study by Cano et al. 9 As the frequency distribution of our ACTS results is skewed, we presented these data as medians and ranges. For our patient population, the mean reverse Burdens score was 56.2 (SD: 4.0; mean Burdens score: 15.8) and the mean Benefits score was 11.6 (SD: 2.5). Treatment satisfaction in other studies was generally lower or similar, with means ranging from 46.5 to 58.7 for the reverse Burdens scale, and from 10.4 to 12.4 for the Benefits scale. 9 26 27 28

Subgroup analysis showed that patients aged 65 and older had a higher treatment satisfaction than younger patients on the Burdens score. Previous studies have also shown that patients 65 years and older are more satisfied with hospital care than younger patients. 29 30 Possible reasons for lower treatment satisfaction in younger patients are higher expectations, 29 higher risk–benefit ratio, and more difficulty to accept chronic anticoagulant treatment.

A possible explanation for the difference in factor structure of the Danish version of the ACTS in comparison to the original version could be a difference in study population. However, we suggest testing of both the two-factor and the four-factor model in future validation studies using CFA.

The internal consistency of the Danish version of the ACTS was in line with previous studies. 9 18 IIC of the Benefits scale was only slightly higher than our predetermined criteria, suggesting that items perhaps are fairly similar. Cano et al also reported a high mean IIC of 0.67 for the Benefits scale. 9 Further rewording of the Benefits scale to prevent concept repetition should be considered in future revisions of the Danish ACTS. Test–retest reliability was good with an ICC of 0.80. Cano et al reported a similar test–retest ICC of 0.79. 9

With regard to the demonstrated ceiling effects, Cano et al suggested that a decrease in response options from five to four and rewording of the response options could improve measurement performance. 9 Another option would be to add an item at the upper end of the scale, to distinguish between high and very high treatment satisfaction. 31 An item with regard to cost could be added. Specifically for dabigatran etexilate, additional items could concern ease of swallowing the capsules and twice daily compared with once daily dosing.

Strengths of the study include application of established quality criteria and guidelines for design and evaluation of the study and sufficient sample size of the total population and for the evaluation of test–retest reliability.

Potential selection bias of participants is a limitation of this study. Respondents were younger, had higher weight and BMI, and better kidney function. The results may therefore not be representative of all atrial fibrillation patients treated with dabigatran etexilate, especially not the older and multimorbid patients. Furthermore, some nonrespondents discontinued dabigatran etexilate treatment before 1 month of follow-up due to side effects, which may have resulted in a lower treatment satisfaction. Inclusion of a group of patients commencing a wider range of anticoagulants would have resulted in higher generalizability and a more diverse range of experiences in relation to treatment burden.

We recommend that the ACTS is revised for the following reasons: (1) the factor structure is unclear and should be based on a conceptual model 32 33 and (2) a substantial ceiling effect hampers the measurement of improvement. The revised ACTS version should also be tested in a longitudinal study assessing hypothesis testing, and testing for responsiveness and interpretation. Validation in different patient populations and clinical settings with an indication for oral anticoagulant treatment should take place.

## Conclusion

The ACTS, a PRO measurement instrument for oral anticoagulant treatment satisfaction, was translated into Danish and culturally adapted, and is now available for use in patient care and clinical studies. Evaluation of psychometric properties of the Danish version of the ACTS in a population of atrial fibrillation patients treated with dabigatran etexilate showed moderate validity with a factor structure which differs from that of the original version and acceptable reliability. Ceiling effects challenge detection of improvement of treatment satisfaction in clinical practice in this patient population.

## References

[1] Kirchhof P Benussi S Kotecha D 2016 ESC Guidelines for the management of atrial fibrillation developed in collaboration with EACTS Eur Heart J 2016 37 38 2893 2962 27567408 10.1093/eurheartj/ehw210

[2] Zoni-Berisso M Lercari F Carazza T Domenicucci S Epidemiology of atrial fibrillation: European perspective Clin Epidemiol 2014 6 213 220 24966695 10.2147/CLEP.S47385 PMC4064952

[3] Mekaj Y H Mekaj A Y Duci S B Miftari E I New oral anticoagulants: their advantages and disadvantages compared with vitamin K antagonists in the prevention and treatment of patients with thromboembolic events Ther Clin Risk Manag 2015 11 967 977 26150723 10.2147/TCRM.S84210 PMC4485791

[4] Bamber L Wang M Y Prins M H Patient-reported treatment satisfaction with oral rivaroxaban versus standard therapy in the treatment of acute symptomatic deep-vein thrombosis Thromb Haemost 2013 110 04 732 741 23846019 10.1160/TH13-03-0243

[5] European Medicines Agency. Summary of opinion (post authorisation). Available at:http://www.ema.europa.eu/docs/en_GB/document_library/Summary_of_opinion/human/000829/WC500105283.pdf. Accessed December 28, 2017

[6] European Medicines Agency. Summary of opinion (post authorisation). Available at:http://www.ema.europa.eu/docs/en_GB/document_library/Summary_of_opinion/human/000829/WC500165674.pdf. Accessed December 28, 2017

[7] Connolly S J Ezekowitz M D Yusuf S Dabigatran versus warfarin in patients with atrial fibrillation N Engl J Med 2009 361 12 1139 1151 19717844 10.1056/NEJMoa0905561

[8] Baumhauer J F Patient-reported outcomes - are they living up to their potential? N Engl J Med 2017 377 01 6 9 28679102 10.1056/NEJMp1702978

[9] Cano S J Lamping D L Bamber L Smith S The Anti-Clot Treatment Scale (ACTS) in clinical trials: cross-cultural validation in venous thromboembolism patients Health Qual Life Outcomes 2012 10 120 23013426 10.1186/1477-7525-10-120 PMC3478969

[10] Ageno W Mantovani L G Haas S Safety and effectiveness of oral rivaroxaban versus standard anticoagulation for the treatment of symptomatic deep-vein thrombosis (XALIA): an international, prospective, non-interventional study Lancet Haematol 2016 3 01 e12 e21 26765643 10.1016/S2352-3026(15)00257-4

[11] RIVER registry – RIVaroxaban evaluation in real life setting. Available at:https://www.hra.nhs.uk/planning-and-improving-research/application-summaries/research-summaries/river-registry-rivaroxaban-evaluation-in-real-life-setting/. Accessed December 28, 2017

[12] Coleman C I Haas S Turpie A G Impact of switching from a vitamin K antagonist to rivaroxaban on satisfaction with anticoagulation therapy: the XANTUS-ACTS substudy Clin Cardiol 2016 39 10 565 569 27362695 10.1002/clc.22565 PMC6490868

[13] Weitz J I Haas S Ageno W Global anticoagulant registry in the field - venous thromboembolism (GARFIELD-VTE). Rationale and design Thromb Haemost 2016 116 06 1172 1179 27656711 10.1160/TH16-04-0335

[14] ePROVIDE-online support for clinical outcome assessments. Available at:https://eprovide.mapi-trust.org/instruments/anti-clot-treatment-scale. Accessed December 28, 2017

[15] Wild D Grove A Martin M Principles of good practice for the translation and cultural adaptation process for patient-reported outcomes (PRO) measures: report of the ISPOR task force for translation and cultural adaptation Value Health 2005 8 02 94 104 15804318 10.1111/j.1524-4733.2005.04054.x

[16] Beaton D E Bombardier C Guillemin F Ferraz M B Guidelines for the process of cross-cultural adaptation of self-report measures Spine 2000 25 24 3186 3191 11124735 10.1097/00007632-200012150-00014

[17] Mokkink L B Terwee C B Patrick D L The COSMIN checklist for assessing the methodological quality of studies on measurement properties of health status measurement instruments: an international Delphi study Qual Life Res 2010 19 04 539 549 20169472 10.1007/s11136-010-9606-8 PMC2852520

[18] Suárez C Pose A Montero-Pérez-Barquero M [Validation of satisfaction questionnaire ACTS in outpatients with atrial fibrillation treated with oral anticoagulants in Spain. ALADIN Study] Med Clin (Barc) 2016 147 05 192 198 27423653 10.1016/j.medcli.2016.05.024

[19] de Vet H Terwee C B Mokkink L B Knol D L Measurement in Medicine Cambridge, UK Cambridge University Press 2011

[20] Lynn M R Determination and quantification of content validity Nurs Res 1986 35 06 382 385 3640358

[21] Hu L Bentler P M Cutoff criteria for fit indexes in covariance structure analysis: conventional criteria versus new alternatives Struct Equ Modeling 1999 6 01 1 55

[22] de Vet H C Terwee C B Knol D L Bouter L M When to use agreement versus reliability measures J Clin Epidemiol 2006 59 10 1033 1039 16980142 10.1016/j.jclinepi.2005.10.015

[23] Shrout P E Fleiss J L Intraclass correlations: uses in assessing rater reliability Psychol Bull 1979 86 02 420 428 18839484 10.1037//0033-2909.86.2.420

[24] Davidson M Keating J L A comparison of five low back disability questionnaires: reliability and responsiveness Phys Ther 2002 82 01 8 24 11784274 10.1093/ptj/82.1.8

[25] Roffi M Patrono C Collet J P 2015 ESC Guidelines for the management of acute coronary syndromes in patients presenting without persistent ST-segment elevation: Task Force for the Management of Acute Coronary Syndromes in Patients Presenting without Persistent ST-Segment Elevation of the European Society of Cardiology (ESC) Eur Heart J 2016 37 03 267 315 26320110 10.1093/eurheartj/ehv320

[26] Contreras Muruaga M DM Vivancos J Reig G Satisfaction, quality of life and perception of patients regarding burdens and benefits of vitamin K antagonists compared with direct oral anticoagulants in patients with nonvalvular atrial fibrillation J Comp Eff Res 2017 6 04 303 312 28353372 10.2217/cer-2016-0078

[27] Prins M H Lensing A W Brighton T A Oral rivaroxaban versus enoxaparin with vitamin K antagonist for the treatment of symptomatic venous thromboembolism in patients with cancer (EINSTEIN-DVT and EINSTEIN-PE): a pooled subgroup analysis of two randomised controlled trials Lancet Haematol 2014 1 01 e37 e46 27030066 10.1016/S2352-3026(14)70018-3

[28] Fumagalli S Cardini F Roberts A T Psychological effects of treatment with new oral anticoagulants in elderly patients with atrial fibrillation: a preliminary report Aging Clin Exp Res 2015 27 01 99 102 24880697 10.1007/s40520-014-0243-x PMC4322215

[29] Jaipaul C K Rosenthal G E Are older patients more satisfied with hospital care than younger patients? J Gen Intern Med 2003 18 01 23 30 12534760 10.1046/j.1525-1497.2003.20114.x PMC1494807

[30] Al-Windi A Predictors of satisfaction with health care: a primary healthcare-based study Qual Prim Care 2005 13 67 74

[31] Terwee C B Bot S D de Boer M R Quality criteria were proposed for measurement properties of health status questionnaires J Clin Epidemiol 2007 60 01 34 42 17161752 10.1016/j.jclinepi.2006.03.012

[32] Stucki G Boonen A Tugwell P Cieza A Boers M The world health organisation international classification of functioning, disability and health: a conceptual model and interface for the OMERACT process J Rheumatol 2007 34 03 600 606 17343306

[33] Wilson I B Cleary P D Linking clinical variables with health-related quality of life. A conceptual model of patient outcomes JAMA 1995 273 01 59 65 7996652

